# Data on incidence of bleeding in patients with atrial fibrillation and advanced liver fibrosis on treatment with vitamin K or non-vitamin K antagonist oral anticoagulants

**DOI:** 10.1016/j.dib.2018.01.109

**Published:** 2018-02-06

**Authors:** Daniele Pastori, Gregory Y.H. Lip, Alessio Farcomeni, Francesco Del Sole, Angela Sciacqua, Francesco Perticone, Rossella Marcucci, Elisa Grifoni, Pasquale Pignatelli, Francesco Violi, ATHERO-AF study group, Mirella Saliola, Marco Antonio Casciaro, Danilo Menichelli, Francesco Cribari, Alberto Paladino, Rony Gingis, Marta Novo, Vittoria Cammisotto, Cristina Nocella, Simona Bartimoccia, Roberto Carnevale, Tiziana Di Stefano, Patrizia Iannucci, Elio Sabbatini, Luigi Anastasio, Joseph Tassone Eliezer

**Affiliations:** a I Clinica Medica, Atherothrombosis Centre, Department of Internal Medicine and Medical Specialties of Sapienza University of Rome, Rome, Italy; b Institute of Cardiovascular Sciences, University of Birmingham, Birmingham, United Kingdom; c Department of Public Health and Infectious Diseases, Sapienza University of Rome, Rome, Italy; d Department of Medical and Surgical Sciences, University Magna Græcia of Catanzaro, Catanzaro, Italy; e Department of Experimental and Clinical Medicine, University of Florence, Florence, Italy

## Abstract

This article contains the data showing the different characteristics of atrial fibrillation (AF) patients treated with vitamin K (VKAs) or non-vitamin K antagonist oral anticoagulants (NOACs) screened for the presence of liver fibrosis (LF) and followed to record the occurrence of bleeding and cardiovascular events (CVEs). A detailed description of major and minor bleedings is provided according to anticoagulant treatment (VKAs vs. NOACs) and to the presence of LF.

Data here reported also show a higher incidence rate of CVEs in VKA-treated patients, but not in those on NOACs. The data are supplemental to our original research article titled “Incidence of bleeding in patients with atrial fibrillation and advanced liver fibrosis on treatment with vitamin K or non-vitamin K antagonists oral anticoagulants” (Pastori et al., 2018) [1].

**Specifications Table**

**Table:** 

Subject area	*Medicine*
More specific subject area	*Cardiology*
Type of data	*Tables and Figures*
How data was acquired	*Post-hoc analysis of a prospective cohort of AF patients treated with oral anticoagulants*
Data format	*Analyzed (Cox regression analysis and Kaplan-Meier survival analysis results)*
Experimental factors	*The relationship between liver fibrosis and bleeding events in Af patients on anticoagulants*
Experimental features	*Liver fibrosis defined by FIB-4 score > 3.25.*
Data source location	*Multicenter study. Patients were recruited from Sapienza University of Rome, Rome, Italy; University Magna Græcia of Catanzaro, Catanzaro, Italy; University of Florence, Florence, Italy.*
Data accessibility	*The data are accessible within the article*
Related research article	*“Incidence of bleeding in patients with atrial fibrillation and advanced liver fibrosis on treatment with vitamin K or non-vitamin K antagonist oral anticoagulants” (Pastori et al., 2018 in press)*[1]

**Value of the data**

• Data presented here provide information about the characteristics of patients treated with VKAs or NOACs.
• Data here presented provide a detailed description of bleeding events according to the presence of liver fibrosis.
• These data for the subgroup analysis on the different risk of CVEs in patients treated with VKAs or NOACs.

## 1 Data

The data presented include clinical and biochemical characteristics of atrial fibrillation (AF) patients treated with vitamin K (VKAs) or non-vitamin K antagonist oral anticoagulants (NOACs) (Table 1). Table 2 reports characteristics of patients experiencing or not a bleeding event.

**Table 1:** Characteristics of patients treated with VKAs or NOACs.

	**VKAs (*n* = 1297)**	**NOACs (*n* = 1033)**	***p* Value**
**Age (years)**	72.7 ± 8.9	77.1 ± 9.1	< 0.001
**Women (%)**	44.1	48.3	0.045
**Current cigarette smokers (%)**	16.0	9.6	< 0.001
**Persistent/permanent atrial fibrillation (%)**	66.4	60.8	0.007
**HAS-BLED score**	1.8 ± 1.0	1.5 ± 0.8	< 0.001
**CHA**_**2**_**DS**_**2**_**-VASc score**	3.0 ± 1.5	3.4 ± 1.4	< 0.001
**Arterial Hypertension (%)**	84.2	88.9	0.001
**Diabetes mellitus (%)**	22.7	24.6	0.301
**Heart failure (%)**	12.7	17.5	0.001
**Previous cerebrovascular events (%)**	13.2	18.3	0.001
**Previous cardiac events (%)**	17.7	18.8	0.515
**Anti-platelet drugs (%)**	11.3	10.8	0.739
**Statins (%)**	39.2	43.3	0.050
**AST (U/l)**	23.6 ± 12.0	22.5 ± 10.0	0.011
**ALT (U/l)**	24.9 ± 15.2	22.9 ± 13.4	0.001
**Haemoglobin (g/dl)**	13.5 ± 1.6	13.4 ± 1.6	0.036
Platelet count (*10^**9**^**/L)**	229.4 ± 72.8	222.2 ± 59.4	0.009
**FIB-4 index**	1.7 ± 1.0	1.8 ± 0.8	0.105

**Table 2:** Clinical characteristics of patients with and without bleeding events.

	**Any bleeding**		***p* Value**
	**No (*n* = 1973)**	**Yes (*n* = 357)**	
**Age (years)**	74.5 ± 9.5	75.6 ± 8.0	0.019
**NOAC treatment (vs. VKA) (%)**	47.5	26.9	< 0.001
**Women (%)**	46.5	42.9	0.205
**Current cigarette smokers (%)**	13.1	14.2	0.551
**Persistent/permanent atrial fibrillation (%)**	64.4	62.0	0.399
**HAS-BLED score**	1.7 ± 0.9	1.9 ± 0.9	< 0.001
**CHA**_**2**_**DS**_**2**_**-VASc score**	3.1 ± 1.5	3.3 ± 1.4	0.042
**Arterial Hypertension (%)**	86.0	87.7	0.452
**Diabetes mellitus (%)**	24.1	20.4	0.136
**Heart failure (%)**	15.0	14.1	0.745
**Previous cerebrovascular events (%)**	15.5	15.1	0.937
**Previous cardiac events (%)**	17.4	22.8	0.017
**Anti-platelet drugs (%)**	11.0	11.8	0.647
**Statins (%)**	40.8	41.9	0.725
**Time in therapeutic range (%)**^a^	59.9 ± 22.9	60.6 ± 19.7	0.606
**AST (U/l)**	23.2 ± 11.2	22.9 ± 11.2	0.730
**ALT (U/l)**	24.2 ± 14.6	23.013.3 ±	0.130
**Haemoglobin (g/dl)**	13.4 ± 1.6	13.5 ± 1.7	0.591
Platelet count (*10^**9**^**/L)**	226.7 ± 66.9	223.4 ± 68.9	0.395

a Only for VKA-treated patients.

A detailed description of major and minor bleedings according to anticoagulant treatment (VKAs vs. NOACs) is reported in Table 3. Table 4 reports major bleedings according to the presence of LF (defined by a FIB-4 score > 3.25) in the all cohort.

**Table 3:** Description of major and minor bleeding events according to anticoagulant treatment.

	***Whole cohort (n = 2330)***	***VKAs (n = 1297)***	***NOACs (n = 1033)***
**Major bleedings**			
Cerebral/Subdural (*n*)	18	14	4
Gastrointestinal (*n*)	20	10	10
Muscular (*n*)	11	11	0
Articular (*n*)	10	9	1
Haematuria (*n*)	9	8	1
Epistaxis with fall in Hb (*n*)	5	5	0
Extended hematoma (*n*)	4	3	1
Respiratory (*n*)	2	2	0
Retroperitoneal (*n*)	2	2	0
Ocular (*n*)	14	13	1
Pericardial (*n*)	1	1	0
Metrorrhagia (*n*)	1	1	0
Decrease of Hb ≥ 2 gr/dl (*n*)	23	1	22
**Total (*****n*****)**	**120 (5.1%)**	**80 (6.2%)**	**40 (3.9%)**

**Minor bleedings**			
Epistaxis (*n*)	65	56	9
Gastrointestinal (*n*)	52	34	18
Conjunctival (*n*)	35	33	2
Haematuria (*n*)	38	24	14
Cutaneous (*n*)	19	16	3
Post-intervention (*n*)	6	4	2
Oral (*n*)	10	9	1
Respiratory (*n*)	5	2	3
Ear (*n*)	2	2	0
Metrorrhagia (*n*)	1	1	0
Decrease of Hb < 2 gr/dl (*n*)	4	0	4
**Total (*****n*****)**	**237 (10.2%)**	**181 (13.9%)**	**56 (5.4%)**

**Table 4:** Major bleedings according to the presence of liver fibrosis in the all cohort.

	**FIB-4 > 3.25 (*n* = 129)**	**FIB-4 ≤ 3.25 (*n* = 2201)**
**Cerebral/Subdural (*n*)**	4 (3.1%)	14 (0.6%)
**Gastrointestinal (*n*)**	3 (2.3%)	17 (0.8%)
**Muscular (*n*)**	0 (0%)	11 (0.5%)
**Articular (*n*)**	0 (0%)	10 (0,4%)
**Haematuria (*n*)**	1 (0.8%)	8 (0.4%)
**Epistaxis with fall in Hb (*n*)**	2 (1.5%)	3 (0.1%)
**Extended hematoma (*n*)**	0 (0%)	1 (0.04%)
**Respiratory (*n*)**	0 (0%)	2 (0.08%)
**Retroperitoneal (*n*)**	0 (0%)	2 (0.08%)
**Ocular (*n*)**	2 (1.5%)	12 (0.5%)
**Pericardial (*n*)**	0 (0%)	1 (0.04%)
**Metrorrhagia (*n*)**	0 (0%)	1 (0.04%)
**Decrease of Hb ≥ 2 gr/dl (*n*)**	0 (0%)	23 (1.0%)
**Cerebral/Subdural (*n*)**	**12 (9.3%)**	**108 (4.9%)**

**Fig. 1:**
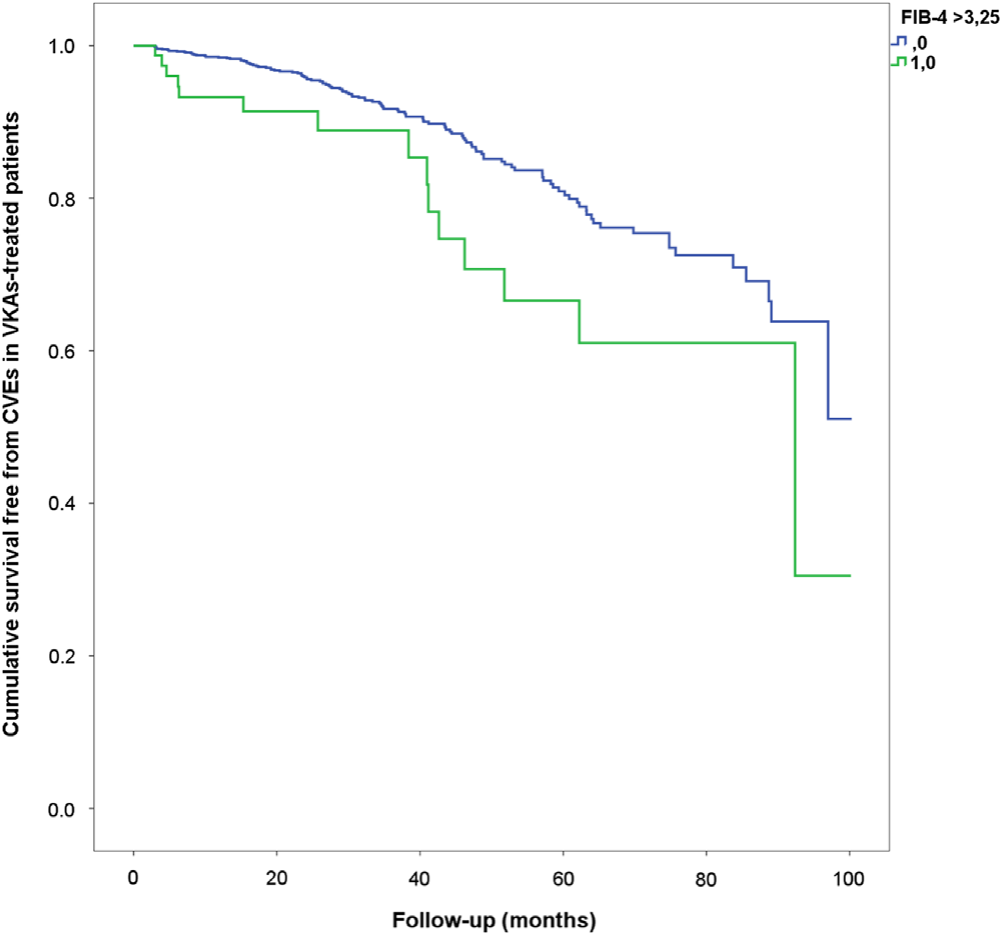
Incidence of CVEs in AF patients treated with VKAs according to FIB-4 value (2.1% vs. 9.8% in patients with and without high FIB-4, log-rank test *p* = 0.005).

**Fig. 2:**
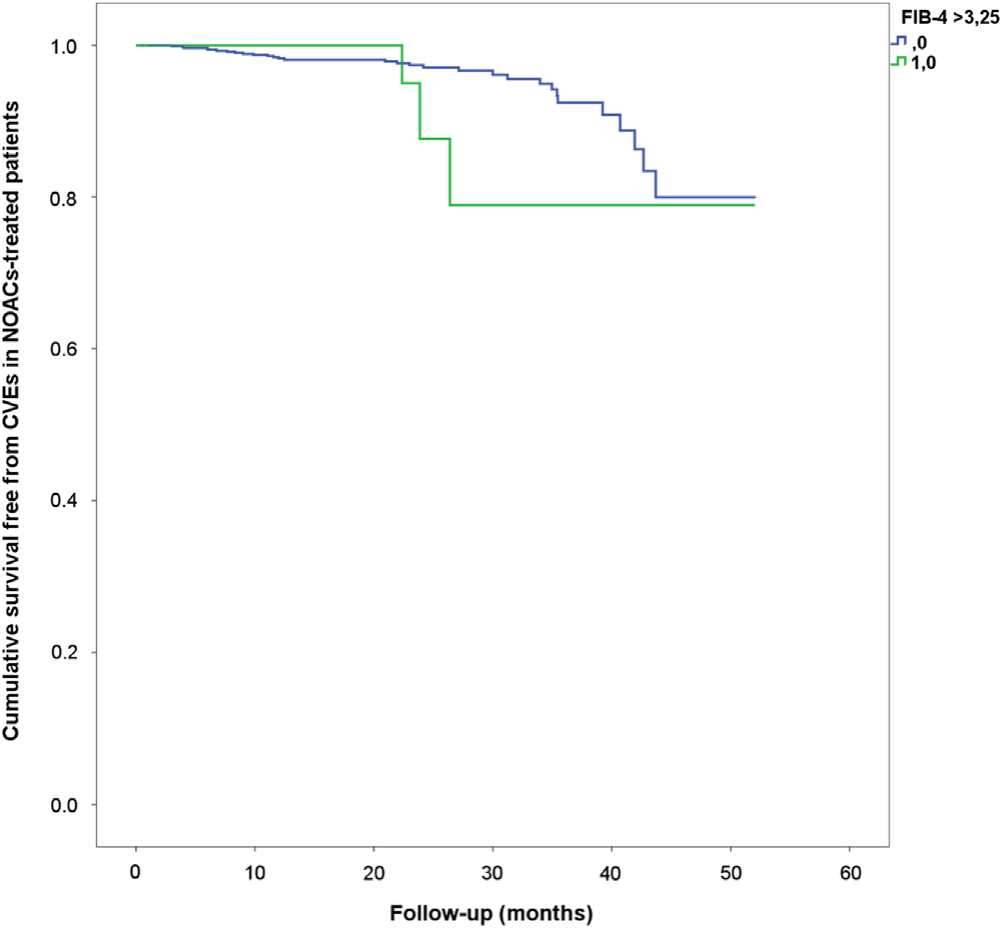
Incidence of CVEs in AF patients treated with NOACs according to FIB-4 value (5.8% vs 3.0% in patients with and without high FIB-4, log-rank test, *p* = 0.279).

Survival analysis showed that in VKA-treated patients with high FIB-4, a higher incidence of cardiovascular events (CVEs) compared to those with normal FIB-4 (2.1% vs. 9.8%, log-rank test *p* = 0.005) was found (Fig. 1). In the NOAC group, a similar rate of CVEs was observed between the two groups (5.8% vs 3.0% in patients with and without high FIB-4, log-rank test, *p* = 0.279, Fig. 2).

## 2 Experimental design, materials, and methods

We performed a post-hoc analysis of a prospective multicentre observational cohort study including 2330 AF patients treated with VKAs (*n* = 1297) or NOACs (*n* = 1033). All patients treated with VKAs (warfarin or acenocoumarol) were locally monitored in specialized anticoagulation clinics for INR determination and VKA prescription. None of the patients measured INRs at home (i.e. with point of care devices) and time in therapeutic range (TTR) was used to assess the quality of anticoagulation according to the linear interpolation method described by Rosendaal et al. [2]. NOACs were prescribed according to the regulatory Italian Agency of Drugs (AIFA) indications and European Society of Cardiology (ESC) guidelines [3].

Exclusion criteria were: prosthetic heart valves, cardiac revascularization in the previous year, severe cognitive impairment, chronic autoimmune systemic diseases, and active cancer. Patients treated with antiplatelet drugs alone were also excluded. At baseline, information about personal medical history and concomitant medications were collected, and HAS-BLED (the labile INR was scored 0 in NOAC users). and CHA_2_DS_2_-VASc scores were calculated. Cardiovascular risk factors, such as arterial hypertension [4], type 2 diabetes mellitus [5] and heart failure [6] were defined according to international guidelines. Patients underwent routine laboratory analyses including AST (U/l), ALT (U/l), haemoglobin (g/dl) and platelet count (× 10^9^/L).

The presence of significant LF was assessed non-invasively by FIB-4 in all patients; FIB-4 was calculated according Sterling et al. by the formula: age (years) × AST (U/L)/PLT (10^9^/L) × ALT (U/L)^1/2^. A value of FIB-4 > 3.25 was set as cut-off for LF [7]. FIB-4 has been validated in different settings of CLD, such viral and metabolic liver disease [8], [9].

### 2.1 Study primary endpoint

Bleeding events were classified according to the International Society on Thrombosis and Hemostasis (ISTH) [10]. Major bleeding was defined as fatal bleeding, symptomatic bleeding in a critical area or organ, i.e. intracranial, intraspinal, intraocular, retroperitoneal, intra-articular, pericardial or intramuscular with compartment syndrome; bleeding causing a fall in haemoglobin level of 2 g/dl or more or leading to transfusion of two or more units of whole blood or red blood cells. All cases of clinically relevant bleeding events that were not classified as major were considered as minor.

### 2.2 Study secondary endpoint

The secondary endpoint was a composite of CVEs including fatal/non-fatal myocardial infarction (MI) or ischemic stroke, cardiac revascularization (stent placement or coronary artery bypass graft), cardiovascular death, transient ischemic attack (TIA) and systemic embolism. Diagnosis of MI was made according to the third universal definition [11]. The occurrence of ischemic stroke was determined on clinical manifestations and confirmed by computed tomography or magnetic resonance; TIA was defined according to the Classification of Cerebrovascular Diseases III [12]. If a patient died within 4 weeks of myocardial infarction or ischemic stroke, these events were recorded as fatal myocardial infarction or ischemic stroke, respectively. Systemic embolism was defined as an acute occlusion of a vessel of an extremity or organ, documented by imaging, surgery, or autopsy findings. Death was classified as cardiovascular unless an unequivocal non-cardiovascular cause of death was identified. Cardiovascular death included sudden death, progressive congestive HF, and procedure-related death.

### 2.3 Statistical analysis

Categorical variables were reported as counts (percentages). The normal distribution of parameters was assessed by Kolmogorov–Smirnov test. Continuous variables were expressed as mean ± standard deviation, or median and interquartile range. Independence of categorical variables was tested with the χ^2^ test. Student *t* test for unpaired samples was used to compare means.

The cumulative incidence of bleedings and CVEs were estimated using a Kaplan–Meier product-limit estimator. Survival curves were formally compared using the log-rank test. Cox proportional hazards analyses were used to calculate the adjusted relative hazard ratio (HR) by each clinical variable. Due to a significant difference in the length of follow-up, a separate survival analysis was performed according to the use of VKAs or NOACs, respectively. Covariates used as candidates for multivariable models included: low TTR (< 70%, only for VKA patients), age, sex, current cigarette smoking, arterial hypertension, diabetes, high FIB-4 (> 3.25), previous cardiac events, previous cardiovascular events, heart failure, haemoglobin, antiplatelet drugs and statins. The final multivariable model was chosen through forward stepwise selection.

Statistical significance was set at a *p* value < 0.05. All tests were two-tailed and analyses were performed using computer software packages (SPSS-18.0, SPSS Inc.).

## References

[1] Pastori D., Lip G.Y.H., Farcomeni A. (2018). Incidence of bleeding in patients with atrial fibrillation and advanced liver fibrosis on treatment with vitamin K or non-vitamin K antagonists oral anticoagulants. Int. J. Cardiol..

[2] Rosendaal F.R., Cannegieter S.C., van der Meer F.J., Briet E. (1993). A method to determine the optimal intensity of oral anticoagulant therapy. Thromb. Haemost..

[3] Kirchhof P., Benussi S., Kotecha D. (2016). 2016 ESC Guidelines for the management of atrial fibrillation developed in collaboration with EACTS: the task force for the management of atrial fibrillation of the European Society of Cardiology (ESC) developed with the special contribution of the European Heart Rhythm Association (EHRA) of the ESC endorsed by the European Stroke Organisation (ESO). Eur. heart J..

[4] Mancia G., Fagard R., Narkiewicz K. (2013). 2013 Practice guidelines for the management of arterial hypertension of the European Society of Hypertension (ESH) and the European Society of Cardiology (ESC): ESH/ESC task force for the management of arterial hypertension. J. Hypertens..

[5] Authors/Task Force M, Ryden L., Grant P.J. (2013). ESC Guidelines on diabetes, pre-diabetes, and cardiovascular diseases developed in collaboration with the EASD: the task force on diabetes, pre-diabetes, and cardiovascular diseases of the European Society of Cardiology (ESC) and developed in collaboration with the European Association for the Study of Diabetes (EASD). Eur. heart J..

[6] McMurray J.J., Adamopoulos S., Anker S.D. (2012). ESC guidelines for the diagnosis and treatment of acute and chronic heart failure 2012: the task force for the diagnosis and treatment of acute and chronic heart failure 2012 of the European Society of Cardiology. Developed in collaboration with the Heart Failure Association (HFA) of the ESC. Eur. J. heart Fail..

[7] Sterling R.K., Lissen E., Clumeck N. (2006). Development of a simple noninvasive index to predict significant fibrosis in patients with HIV/HCV coinfection. Hepatology.

[8] Joo S.K., Kim W., Kim D. (2017). Steatosis severity affects the diagnostic performances of noninvasive fibrosis tests in nonalcoholic fatty liver disease. Liver Int.: Off. J. Int. Assoc. Study Liver.

[9] Li Q., Ren X., Lu C., Li W., Huang Y., Chen L. (2017). Evaluation of APRI and FIB-4 for noninvasive assessment of significant fibrosis and cirrhosis in HBeAg-negative CHB patients with ALT </ = 2 ULN: a retrospective cohort study. Medicine.

[10] Schulman S., Kearon C., Subcommittee on Control of Anticoagulation of the S, Standardization Committee of the International Society on T, Haemostasis (2005). Definition of major bleeding in clinical investigations of antihemostatic medicinal products in non-surgical patients. Journal of thrombosis and haemostasis. JTH.

[11] Thygesen K., Alpert J.S., Jaffe A.S. (2012). Third universal definition of myocardial infarction. J. Am. Coll. Cardiol..

[12] Anon (1990). Special report from the National Institute of Neurological Disorders and Stroke. Classification of cerebrovascular diseases III. Stroke J. Cereb. Circ..

