# Supplementary material for: Data on incidence of bleeding in patients with atrial fibrillation and advanced liver fibrosis on treatment with vitamin K or non-vitamin K antagonist oral anticoagulants
Source: Data Brief. 2018 Feb 6;17:830–6. doi: 10.1016/j.dib.2018.01.109 (PMC5842291; doi:10.1016/j.dib.2018.01.109)
Supplement: Supplementary file 1 — Transparency document [file mmc1.docx]

Conflict of Interest

No potential conflicts of interest to declare.
